# Neurodegeneration and histochemical plasticity in the rat subiculum after kainic acid-induced epilepsy

**DOI:** 10.1186/1471-2210-11-S2-A17

**Published:** 2011-09-05

**Authors:** Meinrad Drexel, Adrian P Preidt, Elke Kirchmair, Günther Sperk

**Affiliations:** 1 Department of Pharmacology, Medical University Innsbruck, 6020 Innsbruck, Austria

## Background

The subiculum, the main output region of the hippocampus, remains largely preserved in human temporal lobe epilepsy (TLE) and therefore may be importantly involved in the generation of epileptic activity arising from the hippocampal region. Our goal was to characterize histopathological and neurochemical changes in the rat subiculum using the kainic acid (KA) model of TLE and to correlate these alterations with data from EEG-recordings.

## Methods

Rats (n = 35) were implanted with biopotential transmitters (EA-F20, DSI) for continuous EEG/video-monitoring. One week later, they were injected with KA (i.p., 10 mg/kg) and developed seizures and an initial status epilepticus (SE). Rats were killed 1, 8, 30 or 90 days after the initial SE and brain histopathology was investigated using immunohistochemistry and *in situ* hybridization for neuropeptides and calcium-binding proteins as markers for different neuronal subpopulations.

## Results

Rats developed spontaneous seizures 3 to 36 days (15 ± 1.5 d) after the initial SE. Neurodegeneration and reactive gliosis were more pronounced in the proximal than in the distal part of the subiculum. The number of parvalbumin (PV)-ir GABAergic interneurons was significantly reduced in the pyramidal cell layer of the subiculum already 24 hrs after KA injection. The decrease in the number of PV-positive neurons in the subiculum correlated with the number of spontaneous seizures subsequently experienced by the rats. Increased (or even *de novo*) expression of neurokinin B (NKB) and NPY mRNA was observed in pyramidal neurons of the subiculum, and fiber labeling for NKB and NPY was increased at late intervals after SE.

## Conclusions

Early degeneration of PV-ir GABAergic basket- and axo-axonic cells may result in decreased inhibition of pyramidal neurons and affects the numbers of spontaneous seizures occurring later. Since NKB has an excitatory action (activating phospholipase C) on NK_3_ receptors, expression of the peptide in principal neurons of the subiculum may contribute to the generation of epileptic seizures. On the other hand, ectopic expression and subsequent release of NPY from axon terminals of pyramidal neurons may inhibit glutamate release by activating presynaptically located Y_2_ receptors and thus exert an anticonvulsive action.

